# Supplementary material for: Cerebello-thalamo-cortical hyperconnectivity as a state-independent functional neural signature for psychosis prediction and characterization
Source: Nat Commun. 2018 Sep 21;9:3836. doi: 10.1038/s41467-018-06350-7 (PMC6155100; doi:10.1038/s41467-018-06350-7)
Supplement: Supplementary file 1 — Supplementary Information [file 41467_2018_6350_MOESM1_ESM.pdf]

SUPPLEMENTARY MATERIALS

Cao et al., Cerebello-thalamo-cortical hyperconnectivity as a state-independent functional neural signature for psychosis prediction and characterization

TABLE OF CONTENTS

Supplementary Methods 2
- Subjects 2
- fMRI paradigms and data acquisition 3
- Data Preprocessing 4
- Network construction and principal component analysis 4
- Network-based statistic 5
- Supplementary analysis with the subsample 6
- Association with gray matter volume 7
- Verification of findings in the CNP sample 7
- Validity of using task regression in the study 8

Supplementary Discussion 9
- Differences between current work and previous work in psychosis CHR 9

Supplementary Figures 10

Supplementary Tables 16

Supplementary References 23

SUPPLEMENTARY METHODS

SUBJECTS

Two independent samples with multi-paradigm fMRI data were used in this study. The discovery sample (the NAPLS-2 sample) consisted of 182 subjects at CHR for psychosis (including 19 subjects who converted to psychosis during clinical follow-up (CHR-C, age 17.63 ± 3.37 years, 12 males) and 163 subjects who did not convert (CHR-NC, age 18.98 ± 4.20 years, 93 males)) and 120 healthy controls (HC, age 20.10 ± 4.73 years, 70 males) as part of the NAPLS-2 project [1] recruited from eight study sites across the United States and Canada: Emory University, Harvard University, University of Calgary, University of California Los Angeles, University of California San Diego, University of North Carolina Chapel Hill, Yale University, and Zucker Hillside Hospital. The included subjects completed a battery of fMRI scans with five different paradigms (resting state, working memory, episodic memory encoding, episodic memory retrieval, emotional face processing) at the initial recruitment point. All participants provided written informed consent for the study. The protocol and consent forms were approved by the institutional review boards at each site.

The participants received the Structured Clinical Interview for Diagnostic and Statistical Manual of Mental Disorders (DSM-IV [2]) and the Structured Interview for Prodromal Syndromes [3] at baseline and at 6-month intervals up to approximately 2 years, and at the point of conversion to psychosis by trained clinicians. At each assessment point, prodromal symptom severity was quantified using the Scale of Prodromal Symptoms (SOPS [3]). The general exclusion criteria included a prior history of neurological disorders, substance dependency in the last six months and IQ < 70 (assessed by the Wechsler Abbreviated Scale of Intelligence [4]). The CHR subjects met the SIPS criteria for a prodromal risk syndrome [3] at baseline after excluding individuals who had ever met DSM-IV criteria for a psychotic disorder [2]. The converters met DSM-IV criteria for an Axis-I psychotic disorder or had at least one fully psychotic symptom at follow-up. Healthy controls were excluded if they met the criteria for a psychotic disorder or prodromal syndromes, or had a first-degree relative with mental illness. Sample details are provided in Supplementary Table 1.

The verification of our findings was performed in an independent case-control sample (the CNP sample). This sample was drawn from the public dataset (UCLA CNP study [5], https://openfmri.org/dataset/ds000030/) consisting of 50 patients with schizophrenia (SZ), 49 patients with bipolar disorder (BD), 43 patients with attention-deficit hyperactivity disorder (ADHD) and 130 healthy controls (HCs). The subjects were recruited from the greater Los Angeles area and provided written informed consent following procedures approved by the Institutional Review Boards at UCLA and the Los Angeles County Department of Mental Health. The participants underwent part or all of the seven fMRI paradigms employed in the cohort (resting state, risk taking, working memory, episodic memory encoding, episodic memory retrieval, stop signal, task switching). Seven HCs and three patients with ADHD were excluded from the analysis due to the loss of T1 images or insufficient fMRI data available (less than two paradigms, for a full exclusion list see Supplementary Table 6), leaving a total of 262 subjects: 50 SZ (age 36.46 ± 8.88 years, 38 males), 49 BD (age 35.29 ± 9.02 years, 28 males), 40 ADHD (age 32.05 ± 10.41 years, 21 males) and 123 HCs (age 31.53 ± 8.80 years, 65 males). Sample details are provided in Supplementary Table 3.

Diagnoses for the three disorders in the sample were based on the structured clinical Interview for DSM-IV [2] and the Adult ADHD Interview [6]. Any patients who met criteria for more than one diagnosis were excluded. Healthy subjects were excluded if they had a life-time diagnosis of a major Axis-I disorder, substance abuse or significant medical illness. For all participants, verbal IQ was quantified using the vocabulary subtest of the Wechsler Adult Intelligence Scale (WAIS [7]), and performance IQ was measured using the matrix reasoning subtest of the WAIS. See Poldrack et al. [5] for a detailed sample description of this public dataset.

fMRI PARADIGMS AND DATA ACQUISITION

The NAPLS-2 sample included five fMRI paradigms: an eyes-open resting state paradigm, a verbal working memory task, a paired-associates memory encoding task, a paired-associates memory retrieval task and an emotional face matching task. Details on these paradigms have been fully described in our previous work [8,

[9, 10]. All data were acquired from 3T MR scanners (Siemens Trio, GE HDx and GE Discovery) located at eight study sites using gradient-recalled-echo echo-planar imaging (GRE-EPI) sequences with identical parameters at all eight sites. The working memory and face matching tasks were scanned with TR = 2.5 s while the other paradigms used TR = 2 s. All other parameters were the same across all paradigms (TE = 30 ms, 77 degree flip angle, 30 4-mm slices, 1mm gap, 220 mm FOV).

The CNP dataset included seven fMRI paradigms: an eyes-open resting state paradigm, a balloon analog risk taking task, a spatial working memory task, a paired-associate memory encoding task, a paired-associate memory retrieval task, a "Go-No Go" stop signal task and a "color-shape" task-switching task. Details on these paradigms see Poldrack et al. [5]. The data were collected from 3T Siemens Trio scanners located at UCLA using the same GRE-EPI sequence: TR = 2s, TE = 30 ms, 90 degree flip angle, 34 4-mm slices, 192 mm FOV.

Data Preprocessing

Both samples followed the same data preprocessing pipelines implemented in the Statistical Parametric Mapping software (SPM12, http://www.fil.ion.ucl.ac.uk/spm/software/spm12/). In brief, all images were slice-time corrected, realigned for head motion, registered to the individual T1-weighted structural images, and spatially normalized to the Montreal Neurological Institute (MNI) template. Finally, the normalized images were spatially smoothed with an 8 mm full-width at half-maximum (FWHM) Gaussian kernel.

Network construction and principal component analysis

The network analysis followed previously published procedures [10, 11, 12, 13] based on a functionally defined brain atlas reported by Power et al [14]. Of note, the original Power atlas with 264 nodes does not include nodes in the bilateral hippocampus, bilateral amygdala and bilateral ventral striatum. Since these regions are of particular interest in clinical neuroscience and in psychotic disorders, we additionally included these nodes based on previously published coordinates

from meta-analyses [15, 16, 17], thereby increasing the total number of nodes to 270 (one node per region and hemisphere). This expanded Power atlas has also been used in prior work [10, 12, 18].

We extracted the mean time series of each of the 270 nodes from the preprocessed images. The extracted time series were further corrected for mean effects of task-evoked coactivations (for task data), white matter (WM) and cerebrospinal (CSF) signals, 24 head motion parameters (6 translation and rotation parameters, their first derivatives, and the square of these 12 parameters), and frame-wise displacement (FD), and then temporally filtered (rest data: band-pass 0.008-0.1 Hz; task data: high-pass 0.008 Hz). Pairwise correlations were performed between the noise-corrected, filtered time series of each of the 270 nodes, yielding a 270 × 270 connectivity matrix for each subject during each paradigm. All connectivity matrices for the same subjects were then vectorized, mean centered and entered into a principal component analysis (PCA) using singular value decomposition (SVD). Essentially, the PCA decomposes the original connectivity matrices into a set of principal components (PCs) that are orthogonal to each other. Each generated component is a linear combination of original matrices, and the components are organized in the way such that the first PC accounts for the largest variance in the original data. In our data, the PCA yielded a total of five PCs, where the first PC scores represent the shared connectivity patterns that explain the most variance across all paradigms. We extracted these first PC scores for each subject for further statistical analysis.

NETWORK-BASED STATISTIC

We first aimed to identify connectome-wide state-independent changes that can potentially predict psychosis. To this end, network-based statistic (NBS) was employed to probe connectivity differences between the three outcome groups in the NAPLS-2 sample. The whole procedure followed the previously published work [11, 12, 19]. In brief, NBS controls for cluster-level family-wise error (FWE) occurred during the entry-wise matrical comparisons and offers a larger power than mass-univariate tests on independent entries. This was done by first applying an initial linear regression model on each of the N(N-1)/2 = 36,315 (N = 270)

edges in the individual-specific PC matrices, with group modeled as regressor of interest. Notably, since the PCA approach essentially extracts the common variance that is shared across paradigms, it is likely to capture signals that are associated with subjects' demographic, head motion, and/or medication status. Therefore, we strictly controlled for these variables in the regression model by including age, sex, IQ, site, mean FD across all paradigms and antipsychotic dosage (measured as chlorpromazine equivalent dosage per day) as regressors. This step generated a *P*-value matrix representing the probability of accepting the null hypothesis on the group effect for each edge. All edges with P values < 0.0005 were then thresholded into a set of suprathreshold links, and connected clusters within this set were identified using breadth first search [20]. The significance of the identified clusters was subsequently tested by 10,000 permutation testing, where subjects were randomly reallocated into the three groups and the maximal size of the identified cluster was recalculated during each permutation. The corrected *P* value for the cluster was determined by the proportion of the derived cluster sizes in the permutation distribution that were larger than the observed group difference.

SUPPLEMENTARY ANALYSIS WITH THE SUBSAMPLE

To further verify that the observed network changes were not driven by demographical, motion and medication differences between groups, we tested the significance of the identified network in an unmedicated and matched subsample. The subsample was selected using an iteration-based approach. First, all subjects that had received any antipsychotic medications or had a mean FD $\geqslant 0.5$ mm were excluded. The remaining subjects in the converter group (11 subjects) were used as the target sample and the remaining subjects in the non-converter and control groups were randomly selected to match this target sample in terms of age, sex, IQ and head motion. The whole procedure was iterated by 10,000 times, and the optimal subsample was chosen for the non-converter and control groups as having the lowest deviation of these measures from the converter group during iterations. An analysis of covariance (ANCOVA) model was subsequently performed, where the mean values of the identified network were extracted from the PC matrices for all selected subjects and entered into the model as dependent

variable and group as independent variable, covarying for age, sex, site, IQ and head motion. Significance was set at $P < 0.05$.

ASSOCIATION WITH GRAY MATTER VOLUME

To examine whether the observed functional changes would relate to their structural differences, we also analyzed subjects' high-resolution T1-weighted imaging data in the NAPLS-2 sample. The images were processed using the standard pipeline implemented in the FreeSurfer software (version 5.3, https://surfer.nmr.mgh.harvard.edu/). In brief, surface-based cortical reconstruction was performed that includes segmentation of the white matter, tessellation of the gray/white matter boundary, and inflation of the folded tessellated surface in each individual [21, 22, 23]. Cortical thickness measures were extracted by calculating the shortest distance from each point on the gray/white boundary to the pial surface at each vertex [24]. Cortical volumes were subsequently calculated as the multiplication of cortical thickness and cortical area at each vertex. The subcortical volumes labeled using an automated, atlas-based, volumetric segmentation procedure [25].

For all involved cortical, subcortical and cerebellar regions in the identified network, we calculated their mean volumes from the processed structural data and correlated these measures with the mean values of the network in the PC matrices using Pearson correlation. FWE correction was used to control for false positives in the multiple comparison.

VERIFICATION OF FINDINGS IN THE CNP SAMPLE

As a proof of concept, we further tested the presence and specificity of the observed network changes in the CNP sample. Here, the data processing for the CNP sample followed exactly the same procedures as that for the NAPLS-2 sample. After computing the first PC scores from the employed paradigms for each individual, the same network that showed significant group effect in the NAPLS-2 sample was extracted from each individual's PC matrix in the CNP sample and was further averaged to generate a subject-specific metric. ANCOVA model was used

to compare the derived metrics between the four groups, where age, sex, IQ, mean FD across all paradigms and antipsychotic dosage were included in the model as covariates. For detected group main effect, post-hoc analyses were performed for pair-wise comparisons between each of the two groups and significant post-hoc findings were considered as $P < 0.05$ after Bonferroni correction. Subsample selection followed the similar procedure as described above, where all subjects with FD > 0.4 mm, performance IQ < 7 or verbal IQ < 5 were removed. The remaining subjects in the converter group (27 subjects) were used as the target sample to match the other three groups by 10,000 iterations, generating an optimally matched subsample for the CNP data.

VALIDITY OF USING TASK REGRESSION IN THE STUDY

The task regression step in the analysis of task-related data raises the question as whether the residual data would simply reflect "resting-state" network structure, in which case the claim of "cross-paradigm connectivity" would be invalid. To examine the validity of this processing step, we performed two supplementary analyses. First, we tested the similarity between the functional connectivity matrices derived from different paradigms and different processing methods (i.e., rest data vs task data without task regression, rest data vs task data with task regression, task data without task regression vs task data with task regression) by computing their Pearson correlations. If the task regression results in a residualized component resembling the resting state, a close similarity between rest data and task data with task regression would be found, which should be higher than the similarity between task data with task regression and task data without task regression. However, the results showed exactly the opposite - the task data with task regression and task data without task regression were highly correlated ($r > 0.97$), and were more similar to each other than task data vs rest data ($r < 0.59$, Supplementary Fig 5). These results support the argument that task regression would not drive the residual data to resemble the resting state.

Second, to assess whether the task regression step would change the main hyperconnectivity findings, we reran the entire analysis using the task data without task regression. Our analysis revealed a very similar network covering the

cerebellum, thalamus and cerebral cortex (P_{FWE} = 0.004, Supplementary Fig 6), suggesting that the observed cerebello-thalamo-cortical hyperconnectivity is not influenced by the task regression procedure.

SUPPLEMENTARY DISCUSSION

DIFFERENCES BETWEEN CURRENT WORK AND PREVIOUS WORK IN PSYCHOSIS CHR

The current work differs from previous work (in particular Anticevic et al. [26]) in terms of both methods and interpretation. First, the analysis in Anticevic et al. was performed on the resting-state data only, and thus the resulting network changes are more likely to reflect *the most significant abnormality in CHR cases during rest*. In contrast, the current work aimed to investigate *the most consistent functional changes in CHR cases, regardless of paradigm*. As a consequence, we interpret the results as a reflective of "state-independent" change rather than a significant change during any specific paradigm. Second, Anticevic's work was hypothesis-driven and specifically tested the connectivity between thalamus and other parts of the brain using the thalamus as a seed region. The current work, however, employed a data-driven method (i.e. NBS) without any a priori hypothesis. Taken together, work by Anticevic et al. was a verification study testing a specific hypothesis during resting state, while the current work is an discovery-oriented study investigating the most consistent changes in CHR independent of paradigm.

Supplementary Figures

Supplementary Fig 1. Percent of variance explained by the first PC (A) and factor loadings for the first PC in each paradigm (B) in the NAPLS-2 sample. Almost 70% of total variance was explained by the first PC for all three groups. No significant group differences were found in percent of variance and factor loadings. CHR-C = converters, CHR-NC = non-converters, HC = healthy controls, RS = resting state, WM = working memory, EMenc = episodic memory encoding, EMret = episodic memory retrieval, FM = emotional face matching. Error bars indicate standard errors.

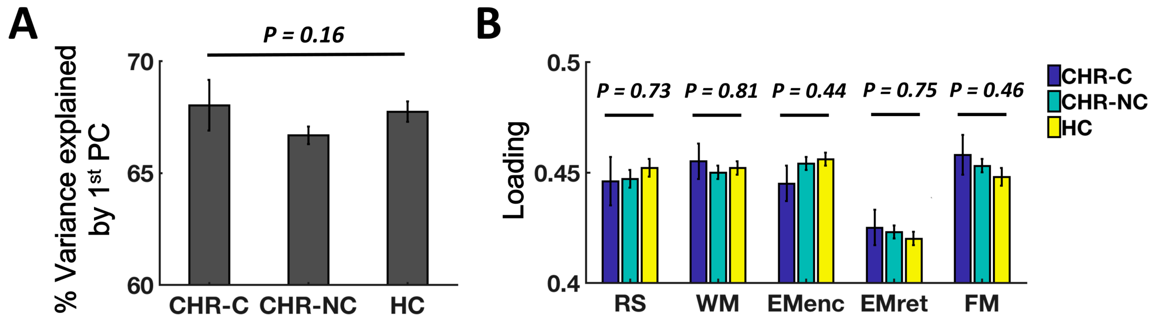

Supplementary Fig 2. Percent of variance explained by the first PC (A) and factor loadings for the first PC in each paradigm (B) in the CNP sample. Similar to the NAPLS-2 sample, almost 70% of total variance was explained by the first PC for the four groups in the CNP sample. No significant group differences were found in percent of variance and factor loadings during the risk taking, episodic memory encoding, episodic memory retrieval and stop signal paradigms. However, the resting state, working memory and task switching paradigms showed a significant difference in factor loadings between groups. RS = resting state, RT = risk taking, WM = working memory, EMenc = episodic memory encoding, EMret = episodic memory retrieval, SS = stop signal, TS = task switching. SZ = schizophrenia, BD = bipolar disorder, ADHD = attention deficit hyperactivity disorder, HC = healthy control. Error bars indicate standard errors.

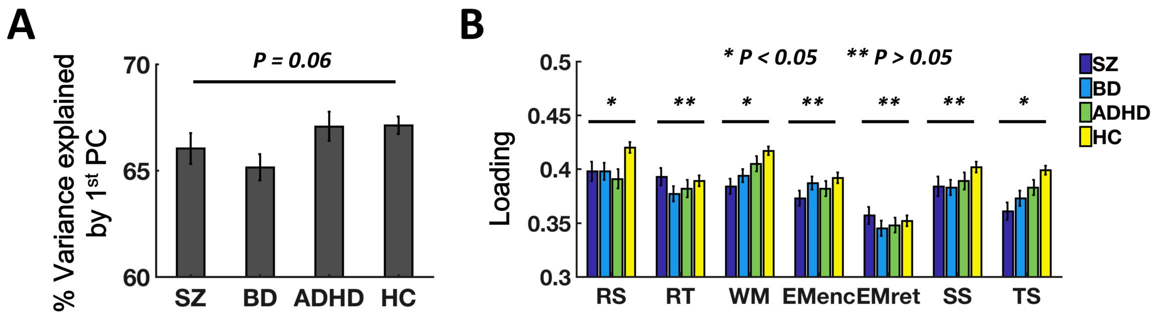

Supplementary Fig 3. Group comparisons of the identified network in the matched subsamples drawn from the NAPLS-2 (A) and CNP (B) samples. For NAPLS-2 subsample, all subjects with medication were removed. Similar to the findings in the larger samples, significant group differences were present in the matched subsamples, suggesting that the observed hyperconnectivity is not driven by group differences in demographics, head motion and/or medication. CHR-C = converters, CHR-NC = non-converters, HC = healthy controls, SZ = schizophrenia, BD = bipolar disorder, ADHD = attention deficit hyperactivity disorder. Error bars indicate standard errors.

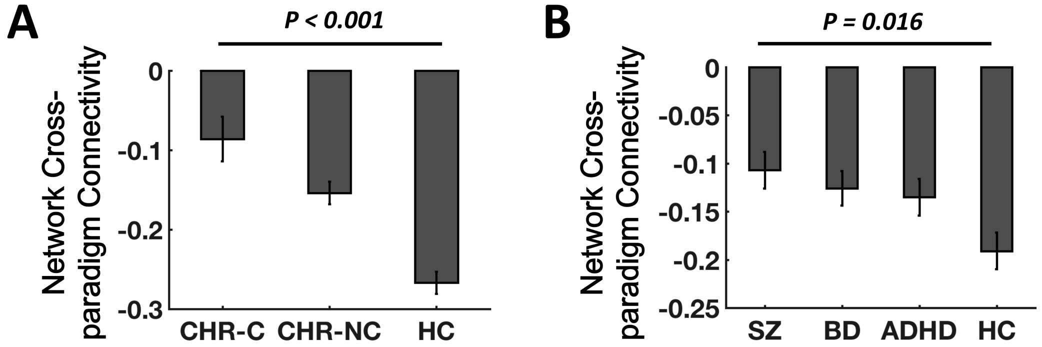

Supplementary Fig 4. Permutation testing on the specificity of the observed cerebello-thalamo-cortical network. For a total of 10,000 permutations (x-axis), none of the derived *P* values were significant after Bonferroni correction. In stark contrast, the observed network (upper right dot) was highly significant even after Bonferroni correction, suggesting the specificity of the observed network in psychosis prediction. The red dashed line indicates the level of significance.

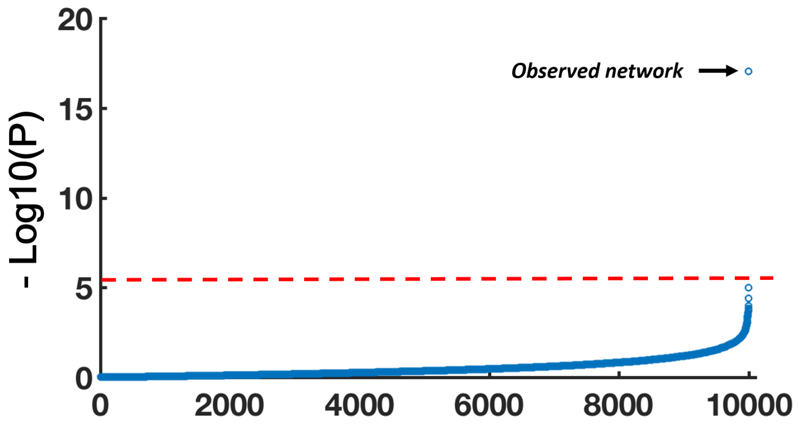

Supplementary Fig 5. Similarity between functional connectivity matrices derived from different paradigms and processing methods. The task data with task regression and task data without task regression were highly correlated, and were much more similar to each other than the rest data vs task data (regardless of task regression), suggesting that task regression would not drive the residual data to resemble the resting state. WM = working memory, EMenc = episodic memory encoding, EMret = episodic memory retrieval, FM = emotional face matching. Error bars indicate standard errors.

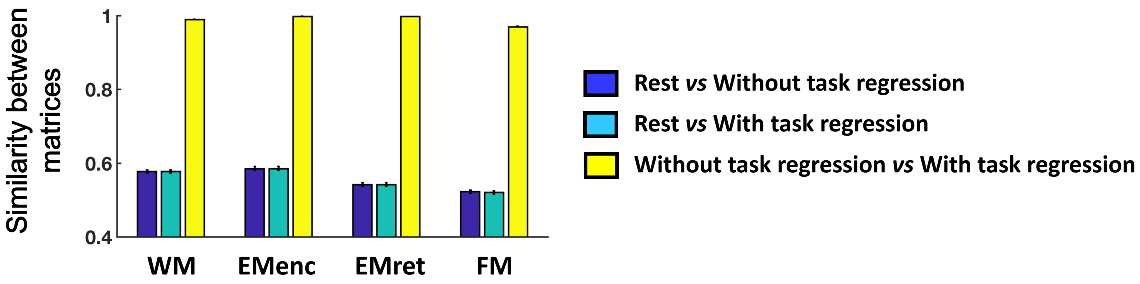

Supplementary Fig 6. Similar cerebello-thalamo-cortical network hyperconnectivity was found using data without task regression. SM = sensorimotor network, VIS = visual network, AUD = auditory network, DMN = default-mode network, FPN = frontoparietal network, CON = cingulo-opercular network, ATT = attention network, SC-CRB = subcortico-cereballar network.

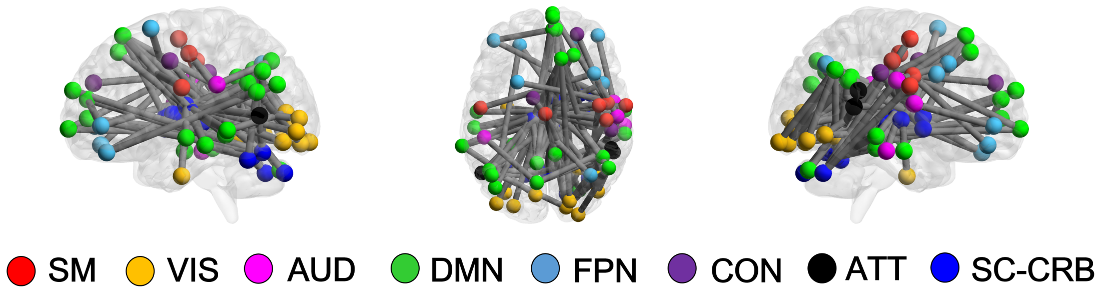

Supplementary Tables

Supplementary Table 1. Demographic and clinical details on the NAPLS-2 sample

	CHR-C (N = 19)	CHR-NC (N = 163)	HC (N = 120)	*P* value
**Age (year)**	17.63 ± 3.37	18.98 ± 4.20	20.10 ± 4.73	Overall: 0.02<br>CHR-C vs. CHR-NC: 0.61
**Sex (M/F)**	12/7	93/70	70/50	0.87
**IQ**	108.63 ± 14.10	104.71 ± 15.20	109.88 ± 14.93	Overall: 0.02<br>CHR-C vs. CHR-NC: 0.85
**SOPS - Positive**	12.47 ± 2.27	11.27 ± 3.77	1.31 ± 2.40	Overall: <0.001<br>CHR-C vs. CHR-NC: 0.37
**SOPS - Negative**	9.74 ± 6.14	10.93 ± 6.07	1.79 ± 2.30	Overall: <0.001<br>CHR-C vs. CHR-NC: 0.96
**SOPS - Disorganization**	7.32 ± 3.86	4.94 ± 3.08	0.76 ± 1.23	Overall: <0.001<br>CHR-C vs. CHR-NC: 0.001
**SOPS - General**	7.74 ± 3.97	8.53 ± 4.32	1.54 ± 2.34	Overall: <0.001<br>CHR-C vs. CHR-NC: 0.99
**Medication (% medicated)**	36.84	19.02	0	Overall: <0.001<br>CHR-C vs. CHR-NC: 0.07
**Medication (CPZ equivalent dosage, mg/day)**	119.87 ± 180.14	36.84 ± 102.31	0	Overall: <0.001<br>CHR-C vs. CHR-NC: <0.001
**Head motion (mean FD, mm)**	0.25 ± 0.15	0.25 ± 0.23	0.19 ± 0.12	Overall: 0.02<br>CHR-C vs. CHR-NC: 0.99

Supplementary Table 2. Demographic and clinical details on the unmedicated, demographically matched subsample drawn from the overall NAPLS-2 sample

	**CHR-C** (N = 11)	**CHR-NC** (N = 40)	**HC** (N = 40)	***P* value**
**Age (year)**	18.36 ± 3.78	19.32 ± 3.58	19.27 ± 4.72	0.78
**Sex (M/F)**	7/4	21/19	24/16	0.71
**IQ**	113.82 ± 12.77	112.75 ± 14.81	113.80 ± 17.02	0.95
**SOPS - Positive**	12.82 ± 2.48	11.28 ± 3.22	1.03 ± 1.72	Overall: <0.001<br>CHR-C vs. CHR-NC: 0.25
**SOPS - Negative**	9.18 ± 7.40	10.05 ± 5.50	1.69 ± 2.35	Overall: <0.001<br>CHR-C vs. CHR-NC: 0.99
**SOPS - Disorganization**	8.00 ± 4.20	4.60 ± 3.02	0.49 ± 0.94	Overall: <0.001<br>CHR-C vs. CHR-NC: 0.001
**SOPS - General**	6.82 ± 4.26	8.75 ± 4.82	1.05 ± 1.62	Overall: <0.001<br>CHR-C vs. CHR-NC: 0.39
**Medication (% medicated)**	0	0	0	-
**Medication (CPZ equivalent dosage, mg/day)**	0	0	0	-
**Head motion (mean FD, mm)**	0.25 ± 0.10	0.17 ± 0.09	0.20 ± 0.12	0.08

Supplementary Table 3. Demographic and clinical details on the CNP sample

	**SZ** (N = 50)	**BD** (N = 49)	**ADHD** (N = 40)	**HC** (N = 123)	***P* value**
**Age (year)**	36.46 ± 8.88	35.29 ± 9.02	32.05 ± 10.41	31.53 ± 8.80	Overall: 0.004<br>SZ vs. HC: 0.01
**Sex (M/F)**	38/12	28/21	21/19	65/58	Overall: 0.04<br>SZ vs. HC: 0.01
**IQ - Performance**	10.04 ± 2.79	13.02 ± 3.01	13.45 ± 2.69	13.52 ± 2.77	Overall: <0.001<br>SZ vs. HC: <0.001
**IQ - Verbal**	7.56 ± 2.17	10.26 ± 2.60	10.72 ± 2.43	10.72 ± 4.86	Overall: <0.001<br>SZ vs. HC: <0.001
**Head motion (mean FD, mm)**	0.27 ± 0.13	0.19 ± 0.78	0.17 ± 0.07	0.15 ± 0.07	Overall: <0.001<br>SZ vs. HC: <0.001
**Medication (CPZ equivalent dosage, mg/day))**	551.00 ± 930.95	252.38 ± 444.77	22.50 ± 118.23	-	<0.001

Supplementary Table 4. Demographic and clinical details on the demographically matched subsample drawn from the overall CNP sample

	**SZ (N = 27)**	**BD (N = 27)**	**ADHD (N = 27)**	**HC (N = 27)**	***P* value**
**Age (year)**	35.93 ± 8.43	36.26 ± 8.29	36.19 ± 10.26	35.59 ± 9.33	0.99
**Sex (M/F)**	18/9	16/11	16/11	14/13	0.75
**IQ - Performance**	11.11 ± 2.22	11.74 ± 2.63	12.85 ± 2.75	12.70 ± 2.80	0.06
**IQ - Verbal**	8.48 ± 1.78	9.15 ± 2.38	10.04 ± 2.38	9.74 ± 2.31	0.06
**Head motion (mean FD, mm)**	0.22 ± 0.07	0.20 ± 0.08	0.18 ± 0.07	0.17 ± 0.09	0.16
**Medication (CPZ equivalent dosage, mg/day))**	411.78 ± 529.36	177.78 ± 308.71	33.33 ± 143.52	-	<0.001

Supplementary Table 5. The 84 edges in the identified network cluster from the NBS analysis in the NAPLS-2 sample

Edge No.	Node A	Node B
1	Occipital_Inf_R	Angular_R
2	Supp_Motor_Area_L	Cerebellum_R
3	Temporal_Mid_L	Lingual_R
4	Angular_R	Lingual_R
5	Frontal_Sup_Medial_R	Lingual_R
6	Frontal_Sup_Medial_R	Lingual_R
7	Angular_R	Lingual_R
8	Angular_R	Occipital_Inf_R
9	Frontal_Sup_Medial_R	Lingual_R
10	Frontal_Med_Orb_R	Temporal_Inf_R
11	Frontal_Sup_Medial_R	Temporal_Inf_R
12	Temporal_Mid_L	Fusiform_R
13	Postcentral_R	Frontal_Sup_Orb_R
14	Occipital_Mid_L	Frontal_Mid_Orb_L
15	Angular_R	Frontal_Mid_Orb_L
16	Angular_R	Frontal_Mid_Orb_L
17	Supp_Motor_Area_L	Cerebellum_R
18	Postcentral_R	Cerebellum_R
19	Precentral_R	Cerebellum_R
20	Postcentral_R	Cerebellum_R
21	Postcentral_R	Cerebellum_R
22	Postcentral_R	Frontal_Mid_R
23	Postcentral_L	Frontal_Mid_R
24	Postcentral_R	Frontal_Mid_R
25	Rolandic_Oper_L	Frontal_Mid_R
26	Rolandic_Oper_R	Frontal_Mid_R
27	Postcentral_R	Frontal_Mid_R
28	Rolandic_Oper_R	Frontal_Mid_Orb_L
29	Frontal_Sup_Medial_R	Frontal_Mid_Orb_L
30	Rolandic_Oper_R	Frontal_Mid_Orb_R
31	Postcentral_R	Thalamus_L
32	Postcentral_R	Thalamus_L
33	Postcentral_R	Thalamus_L
34	Claustrum_R	Thalamus_L
35	Rolandic_Oper_R	Thalamus_L
36	Postcentral_R	Thalamus_L
37	Temporal_Mid_L	Thalamus_L
38	Temporal_Mid_R	Thalamus_L
39	Temporal_Mid_L	Thalamus_L
40	Temporal_Mid_L	Thalamus_L
41	ParaHippocampal_L	Thalamus_L
42	Lingual_R	Thalamus_L
43	Fusiform_R	Thalamus_L

44	Occipital_Inf_R	Thalamus_L
45	Lingual_R	Thalamus_L
46	Temporal_Inf_R	Thalamus_L
47	Occipital_Mid_L	Thalamus_L
48	Occipital_Mid_L	Thalamus_L
49	Occipital_Inf_L	Thalamus_L
50	Precentral_R	Thalamus_L
51	Temporal_Mid_L	Thalamus_L
52	Temporal_Mid_L	Thalamus_L
53	Temporal_Mid_L	Thalamus_R
54	Temporal_Mid_L	Thalamus_R
55	Precuneus_L	Thalamus_R
56	Temporal_Mid_R	Thalamus_R
57	Temporal_Mid_L	Thalamus_R
58	Angular_R	Thalamus_R
59	Frontal_Sup_Medial_R	Putamen_R
60	Frontal_Sup_Medial_R	Putamen_R
61	Precuneus_L	Temporal_Sup_R
62	Precuneus_L	Temporal_Sup_R
63	Thalamus_L	Temporal_Sup_R
64	Temporal_Mid_L	Cerebellum_L
65	Angular_L	Cerebellum_L
66	Frontal_Sup_Medial_R	Cerebellum_L
67	Frontal_Sup_Medial_R	Cerebellum_L
68	Frontal_Sup_Medial_R	Cerebellum_L
69	Temporal_Mid_R	Cerebellum_L
70	Frontal_Sup_Medial_R	Cerebellum_L
71	Angular_R	Cerebellum_L
72	Temporal_Mid_L	Cerebellum_L
73	Temporal_Inf_R	Cerebellum_R
74	Temporal_Mid_L	Cerebellum_R
75	Temporal_Mid_L	Cerebellum_R
76	Angular_R	Cerebellum_R
77	Temporal_Mid_R	Cerebellum_R
78	Frontal_Sup_Medial_R	Cerebellum_R
79	Angular_R	Cerebellum_R
80	Temporal_Mid_L	Cerebellum_R
81	Frontal_Sup_Medial_R	Cerebellum_R
82	Temporal_Sup_R	Cerebellum_R
83	Thalamus_L	Fusiform_L
84	Thalamus_L	Temporal_Mid_L

Supplementary Table 6. List of subjects excluded from the CNP dataset due to the lack of T1 images or insufficient fMRI data available (less than two paradigms).

**Subject ID**	**Group**	**Reason for exclusion**
10971	HC	No T1
10501	HC	No T1
10299	HC	No T1
10428	HC	No T1
11121	HC	No T1
11067	HC	No T1
70035	ADHD	No T1
70036	ADHD	No T1
10193	HC	Only one fMRI paradigm available
70002	ADHD	Only one fMRI paradigm available

SUPPLEMENTARY REFERENCES

1. Addington J*, et al.* North American Prodrome Longitudinal Study (NAPLS 2): overview and recruitment. *Schizophr Res* **142**, 77-82 (2012).

2. First MB, Spitzer RL, M.; G, Williams JBW. Structured Clinical Interview for DSM-IV-TR Axis I Disorders, Research Version, Patient Edition (SCID-I/P). *New York: Biometrics Research, New York State Psychiatric Institute*, (2002).

3. McGlashan TH, Miller TJ, Woods SW, Hoffman RE, Davidson L. Instrument for the Assessment of Prodromal Symptoms and States. In: *Early Intervention in Psychotic Disorders* (ed^(eds Miller T, Mednick SA, McGlashan TH, Libiger J, Johannessen JO). Springer Netherlands (2001).

4. Wechsler D. Wechsler Abbreviated Scale of Intelligence. *Psychological Corporation, New York, NY*, (1999).

5. Poldrack RA*, et al.* A phenome-wide examination of neural and cognitive function. *Sci Data* **3**, 160110 (2016).

6. Kaufman J, Birmaher B, Brent DA, Ryan ND, Rao U. K-Sads-Pl. *Journal of the American Academy of Child and Adolescent Psychiatry* **39**, 1208 (2000).

7. Wechsler D. Wechsler Adult Intelligence Scale - Third Edition (WAIS-III). *The Psychological Corporation*, (1997).

8. Forsyth JK*, et al.* Reliability of functional magnetic resonance imaging activation during working memory in a multi-site study: analysis from the North American Prodrome Longitudinal Study. *Neuroimage* **97**, 41-52 (2014).

9. Gee DG*, et al.* Reliability of an fMRI paradigm for emotional processing in a multisite longitudinal study. *Hum Brain Mapp* **36**, 2558-2579 (2015).

10. Cao H*, et al.* Toward Leveraging Human Connectomic Data in Large Consortia: Generalizability of fMRI-Based Brain Graphs Across Sites, Sessions, and Paradigms. *Cereb Cortex*, (2018).

11. Cao H*, et al.* Altered Functional Subnetwork During Emotional Face Processing: A Potential Intermediate Phenotype for Schizophrenia. *JAMA psychiatry* **73**, 598-605 (2016).

12. Cao H*, et al.* The 5-HTTLPR Polymorphism Affects Network-Based Functional Connectivity in the Visual-Limbic System in Healthy Adults. *Neuropsychopharmacology : official publication of the American College of Neuropsychopharmacology*, (2017).

13. Cao H*, et al.* Test-retest reliability of fMRI-based graph theoretical properties during working memory, emotion processing, and resting state. *Neuroimage* **84**, 888-900 (2014).

14. Power JD*, et al.* Functional network organization of the human brain. *Neuron* **72**, 665-678 (2011).

15. Spreng RN, Mar RA, Kim AS. The common neural basis of autobiographical memory, prospection, navigation, theory of mind, and the default mode: a quantitative meta-analysis. *Journal of cognitive neuroscience* **21**, 489-510 (2009).

16. Sabatinelli D*, et al.* Emotional perception: meta-analyses of face and natural scene processing. *Neuroimage* **54**, 2524-2533 (2011).

17. Liu X, Hairston J, Schrier M, Fan J. Common and distinct networks underlying reward valence and processing stages: a meta-analysis of functional neuroimaging studies. *Neurosci Biobehav Rev* **35**, 1219-1236 (2011).

18. Braun U*, et al.* Dynamic reconfiguration of frontal brain networks during executive cognition in humans. *Proc Natl Acad Sci U S A* **112**, 11678-11683 (2015).

19. Zalesky A, Fornito A, Bullmore ET. Network-based statistic: identifying differences in brain networks. *Neuroimage* **53**, 1197-1207 (2010).

20. Ahuja RK, Magnanti TL, Orlin JB. *Network flows: theory, algorithms, and applications*. Prentice-Hall, Inc. (1993).

21. Dale AM, Fischl B, Sereno MI. Cortical surface-based analysis. I. Segmentation and surface reconstruction. *Neuroimage* **9**, 179-194 (1999).

22. Fischl B, Sereno MI, Dale AM. Cortical surface-based analysis. II: Inflation, flattening, and a surface-based coordinate system. *Neuroimage* **9**, 195-207 (1999).

23. Fischl B*, et al.* Automatically parcellating the human cerebral cortex. *Cereb Cortex* **14**, 11-22 (2004).

24. Fischl B, Dale AM. Measuring the thickness of the human cerebral cortex from magnetic resonance images. *Proceedings of the National Academy of Sciences of the United States of America* **97**, 11050-11055 (2000).

25. Fischl B*, et al.* Whole brain segmentation: automated labeling of neuroanatomical structures in the human brain. *Neuron* **33**, 341-355 (2002).

26. Anticevic A*, et al.* Association of Thalamic Dysconnectivity and Conversion to Psychosis in Youth and Young Adults at Elevated Clinical Risk. *JAMA psychiatry* **72**, 882-891 (2015).
